# [^18^F]FDG PET/CT of Langerhans Cell Histiocytosis with Vertebra Plana

**DOI:** 10.3390/diagnostics15070862

**Published:** 2025-03-28

**Authors:** Tilman Speicher, Moritz B. Bastian, Konstantinos Christofyllakis, Florian Rosar, Samer Ezziddin, Caroline Burgard

**Affiliations:** 1 Department of Nuclear Medicine, Saarland University, 66421 Homburg, Germany; 2 Department of Hematologic Oncology, Saarland University, 66421 Homburg, Germany

**Keywords:** Langerhans cell histiocytosis, FDG, PET/CT, glucose metabolism

## Abstract

We present an ^18^F-fluorodeoxyglucose ([^18^F]FDG) positron emission tomography/computed tomography (PET/CT) scan of a 27 y/o patient with long-standing significant B symptoms, diffuse bone pain, increased inflammation parameters, and polydipsia revealing multiple FDG-avid osteolytic lesions of the axial skeleton including a vertebra plana of T7 and paraosseous soft tissue lesions. A CT-guided biopsy confirmed the diagnosis of Langerhans cell histiocytosis (LCH). This case highlights the importance of considering LCH in young patients with vertebral collapse and underscores the role of PET/CT imaging in establishing an accurate diagnosis.

**Figure 1:**
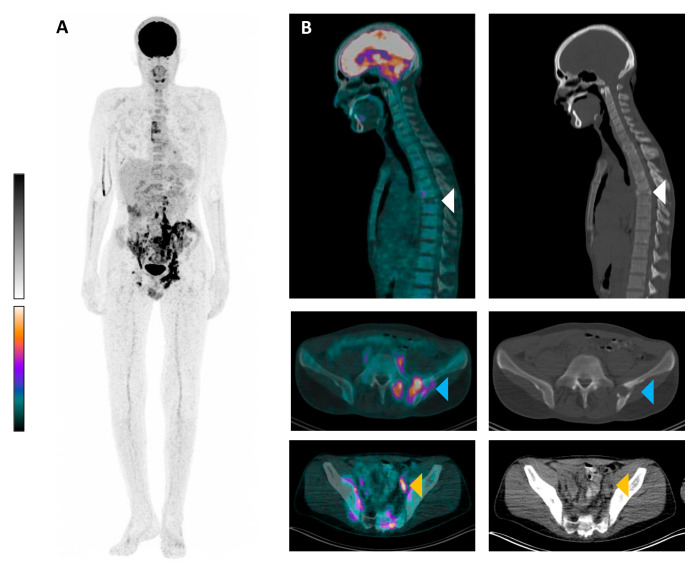
An ^18^F-fluorodeoxyglucose ([^18^F]FDG) positron emission tomography/computed tomography (PET/CT) scan is presented of a 27-year-old man with multifocal FDG-positive manifestations of Langerhans cell histiocytosis (LCH) in bone and lymph nodes as well as paraosseous lesions. The patient initially presented with long-standing significant B symptoms, diffuse bone pain, increased inflammation parameters, and polydipsia with suspected diabetes insipidus. Due to a long-standing, uncharacteristic clinical presentation, LCH was not initially recognized. The assessment of pituitary hormone status was also not conclusive. [^18^F]FDG PET/CT (acquired 69 min post injection of 176 MBq) was performed to further clarify a malignant or inflammatory process (e.g., sarcoidosis). (**A**): maximum intensity projection (MIP); and (**B**): exemplary transversal and sagittal slices of [^18^F]FDG PET/CT showing low glucose metabolism in a vertebra plana of T7 (top row, white arrow heads), intense FDG-positive osseous and paraosseous lesions in the left ilium bone (middle row, blue arrow heads, SUV_max_ 14.7), and intense [^18^F]FDG uptake in a left external iliac lymph node (bottom row, orange arrow heads, SUV_max_ 17.9). Several diseases were considered in the differential diagnosis, including malignant lymphoma, sarcoidosis, and LCH, which was considered the most likely diagnosis based on the vertebra plana. In the literature, it is discussed whether a delayed [^18^F]FDG PET/CT scan can be performed to differentiate between malignant and inflammatory lesions [1,2,3]. Since a biopsy would have been necessary in this case anyway, we decided to forgo the delayed scan. A paraosseous [^18^F]FDG-positive lesion adjacent to the os sacrum on the left was subsequently biopsied under CT guidance ((**B**), blue arrow heads). Histopathological examination confirmed LCH with CD1a-positive-cell proliferation and the detection of a BRAF V600(D/E) mutation. LCH is a rare disorder characterized by the clonal proliferation of Langerhans cells, which are specialized dendritic cells involved in immune regulation [4]. The disease can affect patients of all ages but is most commonly diagnosed in children [5]. LCH presents with a highly variable clinical spectrum, ranging from localized single-organ involvement to multisystem disease with life-threatening complications. Skeletal involvement is the most frequent manifestation, occurring in up to 80% of cases [6]. The axial skeleton, particularly the skull, spine, pelvis, and long bones, is commonly affected. One hallmark radiological finding in LCH of the spine is vertebra plana, a near-complete collapse of the vertebral body, often with preservation of the posterior elements [7]. While vertebra plana can result from various etiologies, such as trauma, infection, or malignancies, LCH is a key differential diagnosis, especially in pediatric patients. Diagnosis is established through imaging, biopsy, and immunohistochemical staining, demonstrating CD1a and Langerin (CD207) positivity [8]. BRAF V600E mutation is the most common genetic driver of LCH [9]. Treatment depends on disease severity, ranging from observation and local therapy in mild cases to systemic chemotherapy, such as cytarabine-based regimens, in multisystem disease [10]. Prognosis varies but is generally favorable in localized forms, with spontaneous bone regeneration frequently observed in vertebra plana. FDG PET/CT represents a key component in the diagnostic process [7,11,12] and is useful in selecting the optimal biopsy site. Wu et al. conducted a study on a cohort of 57 patients with LCH. All patients had at least one FDG-positive lesion suspected to be associated with LCH [13]. The vertebral lesion exhibits low [^18^F]FDG uptake, whereas other bone lesions and lymph node lesions show high [^18^F]FDG uptake. A possible explanation for this could be that the spinal lesions were originally [^18^F]FDG-avid but are no longer active in the course of the disease. Only the collapsed vertebral body remains visible. This case is intended to remind colleagues to consider LCH as a differential diagnosis in the presence of FDG-positive findings and vertebra plana and underscores the role of imaging in establishing an accurate diagnosis.

## Data Availability

The datasets used and analyzed in this paper are available from the corresponding author on reasonable request.
